# Effects of local anesthetics on breast cancer cell viability and migration

**DOI:** 10.1186/s12885-018-4576-2

**Published:** 2018-06-19

**Authors:** Ru Li, Chunyun Xiao, Hengrui Liu, Yujie Huang, James P. Dilger, Jun Lin

**Affiliations:** 1 Department of Anesthesiology, Stony Brook University, Stony Brook, NY USA; 2 Department of Physiology and Biophysics, Stony Brook University, Stony, Brook, NY USA; 3 School of Dental Medicine, Stony Brook University, Stony Brook, NY USA; 4 HSC L4-060, Stony Brook University Health Science Center, Stony Brook, NY 11794-8480 USA

**Keywords:** Local anesthetics, Breast Cancer cells, Cell viability, Cell migration, Cell cycle

## Abstract

**Background:**

Breast cancer accounts for nearly a quarter of all cancers in women worldwide, and more than 90% of women diagnosed with breast cancer undergo mastectomy or breast-conserving surgery. Retrospective clinical studies have suggested that use of regional anesthesia leads to improved patient outcomes. Laboratory studies have reported that breast cancer cells are inhibited by some local anesthetics at millimolar concentration. Here, we present a comprehensive analysis of the effects of six common local anesthetics on two human breast cancer cell lines. We used concentrations ranging from those corresponding to plasma levels during regional block by local anesthetic (plasma concentration) to those corresponding to direct infiltration of local anesthetic.

**Methods:**

Human breast cancer cell lines, MDA-MB-231 and MCF7, were incubated with each of six local anesthetics (lidocaine, mepivacaine, ropivacaine, bupivacaine, levobupivacaine, and chloroprocaine) (10 μM ~ 10 mM) for 6 to 72 h. Assays for cell viability, cytotoxicity, migration, and cell cycle were performed.

**Results:**

High concentrations (> 1 mM) of local anesthetics applied to either MDA-MB-231 or MCF7 cells for 48 h significantly inhibited cell viability and induced cytotoxicity. At plasma concentrations (~ 10 μM) for 72 h, none of the local anesthetics affected cell viability or migration in either cell line. However, at 10 × plasma concentrations, 72-h exposure to bupivacaine, levobupivacaine or chloroprocaine inhibited the viability of MDA-MB-231 cells by > 40% (*p* < 0.001). Levobupivacaine also inhibited the viability of MCF7 cells by 50% (p < 0.001). None of the local anesthetics affected the viability of a non-cancerous breast cell line, MCF10A. MDA-MB-231 cell migration was inhibited by 10 × plasma concentrations of levobupivacaine, ropivacaine or chloroprocaine and MCF7 cell migration was inhibited by mepivacaine and levobupivacaine (*p* < 0.05). Cell cycle analysis showed that the local anesthetics arrest MDA-MB-231 cells in the S phase at both 1 × and 10 × plasma concentrations.

**Conclusions:**

Local anesthetics at high concentrations significantly inhibited breast cancer cell survival. At 10 × plasma concentrations, the effect of local anesthetics on cancer cell viability and migration depended on the exposure time, specific local anesthetic, specific measurement endpoint and specific cell line.

## Background

Breast cancer is one of the most common types of cancer and the second leading cause of cancer death in women. Surgical resection of the primary tumor is the central aspect of the current multiple modes of treatment and has been associated with better prognosis**.** However, recurrence at the primary site or in distant organs does occur and is the major cause of mortality. In fact, the process of surgery, including anesthetic regimens, has increasingly been recognized to affect caner recurrence and metastasis [1]. In clinical practice, surgery for breast cancer may be performed under general anesthesia with or without regional anesthesia. The addition of regional anesthesia in the form of a paravertebral block has been shown to be associated with a longer recurrence free period for patients with breast cancers following surgical resection [2]. Recent retrospective studies have also shown that regional anesthesia improved patient outcome after surgery for other cancers [2, 3]. In addition, the involvement of local anesthetics perioperatively and postoperatively could reduce the use of systemic opioid for pain management [4]. Large-scale prospective clinical studies are currently ongoing to further investigate the potential benefit of local anesthetics [2].

There may be multiple reasons for regional anesthetic-induced benefits leading to less cancer recurrence. One possibility is that the local anesthetics have direct inhibitory effects on the proliferation or migration of cancer cells. Surgical manipulation releases cancer cells into bloodstream [5], which could either seed a recurrence at the primary site or metastasize in distant organs [6]. Meanwhile, local anesthetics are absorbed from injection site to circulation system, where they may encounter circulating cancer cells and affect them. One could even consider perioperative intravenous injection of the local anesthetic lidocaine, at an anti-arrhythmic dose if this concentration proved to be effective in suppressing cancer cells. Alternatively, the surrounding tissue of tumor could be infiltrated with local anesthetic at the concentration range of clinical preparations. Therefore, it is important to determine the direct influence of local anesthetics on cancer cells. However, a comprehensive evaluation of the commonly available local anesthetics on breast cancer cell viability and migration is still lacking.

Here, we evaluated the effects of six common local anesthetics (lidocaine, mepivacaine, ropivacaine, bupivacaine, levobupivacaine, and chloroprocaine) on viability and migration of two well-characterized human breast cancer cell lines MDA-MB-231, MCF-7, and a non-tumorigenic human breast epithelial cell line MCF-10A as a control. First, we examined concentrations corresponding to direct regional infiltration of local anesthetic to a maximum of 10 mM. We then evaluated the effects of lidocaine at anti-arrhythmic dose (10 μM) [7, 8], and other local anesthetics at equipotent nerve block concentrations to lidocaine [9, 10]. These concentrations correspond to the plasma concentrations following regional block and are referred as “plasma concentration” in this paper. For a relative complete range of clinical concentrations, we also utilized 10 times of the plasma concentrations of each local anesthetic, which corresponds to blockage of tetrodotoxin-resistant sodium channels [11]. The information about their potency and efficacy against breast cancer cells would help explain the mechanism of regional anesthesia as well as guide the appropriate selection of local anesthetics and route of the administration.

## Methods

### Cell culture and concentrations of local anesthetics

MDA-MB-231 (ATCC-HTB-26), MCF-7 (ATCC-HTB-22) and MCF-10A (ATCC-CRL-10317) were obtained from ATCC®. MDA-MB-231 cells and MCF-7 cells were cultured in DMEM with 10% FBS and 2% pen/strep. MCF-10A cells were cultured in MEGM mammary epithelial cell growth medium along with additives obtained from Lonza Corporation as a kit (CC-3150). The final culture medium replaced the GA-100 provided with kit to 100 ng/mL cholera toxin.

In the first set of experiments, the cells were treated with high concentrations ranging from 0.3 mM to 10 mM of each local anesthetic, which correspond to direct local infiltration of local anesthetic. In the second set of experiments, the cells were treated with lidocaine at its anti-arrhythmia plasma concentration (10 μM) and approximately equipotent nerve block concentrations for the other anesthetics: 10 μM mepivacaine, 2.8 μM bupivacaine, 2.5 μM levobupivacaine, 3.5 μM ropivacaine, and 15 μM chloroprocaine. In this paper, we refer these concentrations as “plasma concentrations” (Table 1). For a relative complete range of clinical concentrations, we also utilized 10 times of the plasma concentrations of each local anesthetic.

**Table 1:** Clinically relevant concentrations of the local anesthetics used in this study

Local Anesthetics	“Plasma” Concentration (μM)	Local infiltration concentration (μM)
Lidocaine	10	17,500 (0.5%)
Mepivacaine	10	40,592 (1%)
Bupivacaine	2.8	8667 (0.25%)
Levobupivacaine	2.5	8667 (0.25%)
Ropivacaine	3.5	7288 (0.2%)
Chloroprocaine	15	34,670 (1%)

### Cell viability and cell toxicity

Cells were plated at a concentration of 15,000 cells/ml in 96-well plates. For short-term (6 to 24 h) exposure experiments, the local anesthetic was added after the cells reached approximately 70% confluency. For long-term (48 to 72 h) exposure experiments, local anesthetics were added 24 h after cells being plated. Cell viability was assessed using the MTT assay. Viability was calculated from the ratio of absorbance at 571 nm in the drug-treated cells to the drug-free control.

Cell toxicity was evaluated after 48 h of exposure to high doses of local anesthetics using the LDH assay (Roche, Branford, CT) according to manufacturer’s instructions. Briefly, at the end of treatment, three wells with untreated cells were used to determine the maximum LDH release by adding 10 μL 10% Triton X-100 and incubating for 45 min at 37 °C. Then, 50 μL of culture supernatant from each well were transferred to a new 96-well plate and 50 μL of 2X LDH assay buffer was added. The plate was incubated in the dark at room temperature for 30 min. Afterward, a 50 μL stop solution was added to the well, and absorbance at 492 nm was measured.

The cytotoxicity (%) was calculated as (experiment value – medium only control)/(maximum release – medium only control).

### Cell death assay

The apoptosis of cancer cells was assessed after 48 h of exposure to local anesthetics with concentrations ranging from 0.3 mM to 10 mM. MDA-MB-231 or MCF7 cells were seeded into 24 well plates at 1 × 10^5^/well. Cells were harvested using trypsin-EDTA after treatments, and apoptosis assays were performed using Cell Death Detection ELISA^Plus^ (Roche, Cat No. 11920685001, Indianapolis, IN), which is based on the quantitative sandwich enzyme immunoassay using mouse monoclonal antibodies directed against DNA and histones.

### Cell migration assay

Cell migration was assessed after 8 h, 24 h, and 48 h of exposure to local anesthetic using a wound-healing assay. When cells reached more than 90% confluency in 24-well plates, a 200-μL pipet tip was used to scratch a “wound” in the monolayer. The wound will “heal” only if cells migrate along the plate and cover the wound. Images were taken after 0 h, 8 h, 24 h, and 48 h’ incubation with local anesthetics. The wound area in each image was analyzed by the software Image J. Results were calculated as (remaining wound area) / (wound area at 0 h).

### Cell cycle analysis

Cell cycle was analyzed with flow cytometry and propidium iodide. After treatments, cells were washed with cold PBS, and resuspended at 1 × 10^6^/mL. Cells were fixed by adding an equal volume of cold absolute ethanol and were incubated for at least two hours at 4 °C. Cells were washed with cold PBS, and stained with propidium iodide (0.1% Triton X-100, 0.2 mg/mL DNAse-free RNAse A, 0.02 mg/mL in cold PBS) at 37 °C for 15 min. BD FACSCalibur was used to acquire data, which was then analyzed by FlowJo (Version 9.3.2) using Dean-Jett-Fox fit.

### Statistics

Experiments were repeated three times. Means and standard deviations are shown in the figures. ANOVA was used to assess significance (*p* < 0.05). Dunnett’s post hoc tests were used to test difference between groups. GraphPad Prism (version 6) was used to calculate statistics.

## Results

### Viability of MDA-MB-231, MCF-7, and MCF10A cells treated with high doses of local anesthetics

**Fig. 1:**
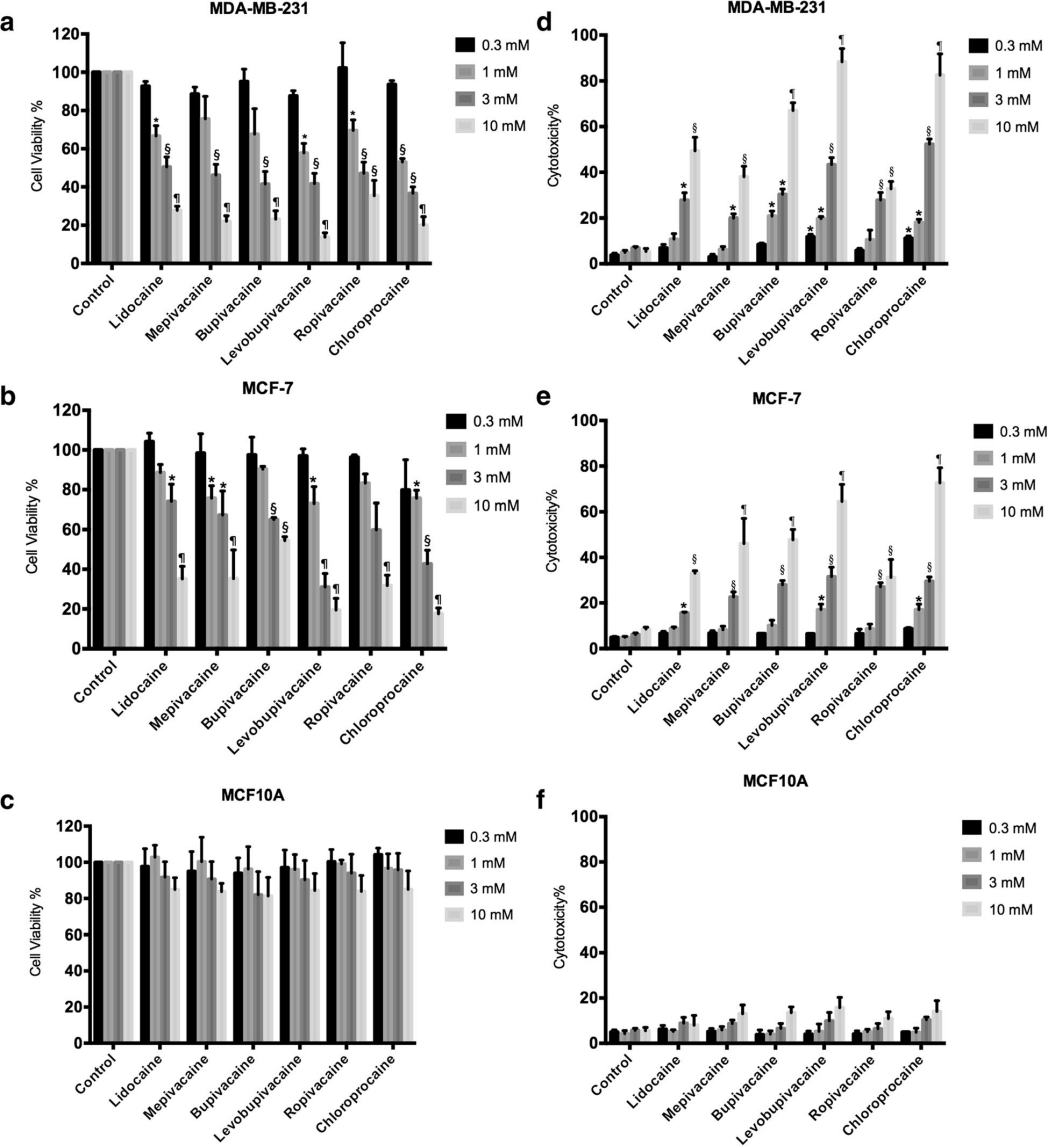
The effect of high concentrations of local anesthetics. Viability was measured by the MTT assay for **a** MDA-MB-231, **b** MCF-7, and **c** MCF10A cells after 48 h exposure to the indicated local anesthetic. The LDH assay was performed after 48 h of exposure to the indicated local anesthetic. **d** MDA-MB-231 cells. **e** MCF-7 cells. **f** MCF10A cells (Significant differences from control cells are indicated by *p < 0.05, §*p* < 0.01, ¶*p* < 0.001)

To establish the response of cancer cells treated with clinical preparation concentrations of local anesthetics, we exposed cells to concentrations ranging from 0.3 mM (30 times higher than the anti-arrhythmia plasma concentration of lidocaine) to 10 mM. After 48 h, we performed MTT assays to assess cell viability. With MDA-MB-231 cells, all local anesthetics at or above 3 mM resulted in more than 40% cell death (Fig. 1a). Three of the local anesthetics at 1 mM concentration, lidocaine, levobupivacaine and chloroprocaine caused 30% cell death. MCF-7 cells showed a similar response. Significant cell death was caused by 1 mM mepivicaine, levobupivacaine and chloroprocaine only but higher concentrations of all local anesthetics were effective at killing cells (Fig. 1b). LDH assays showed results that are consistent with the MTT assays. For MDA-MB-231 cells, all six local anesthetics induced significant cellular toxicity at concentrations higher than 1 mM (Fig. 1d). For MCF-7 cells, similar results were observed (Fig. 1e). Local anesthetics at millimolar concentrations did not affect viability (Fig. 1c) or cellular toxicity (Fig. 1f) of non-tumorigenic human breast epithelial MCF10A cells.

**Fig. 2:**
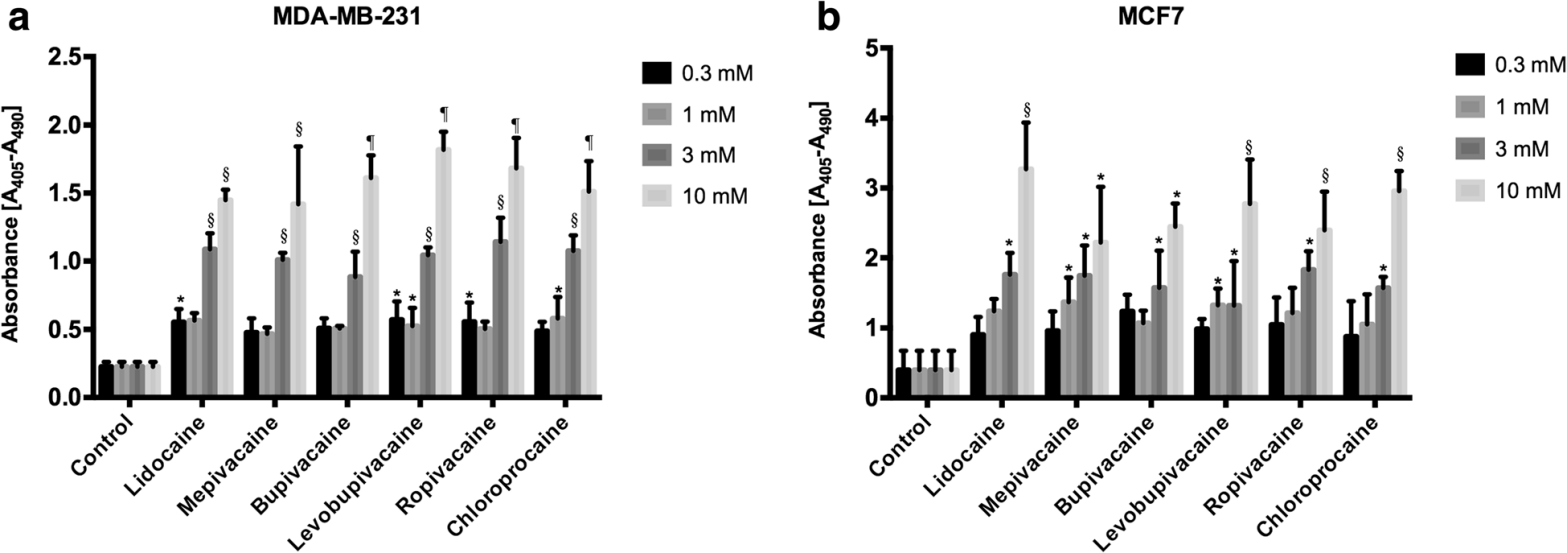
Apoptotic effect of local anesthetics at high concentrations on breast cancer cells. The cell death ELISA assay was carried out after 48 h of exposure to the indicated local anesthetic. **a** MDA-MB-231 cells. **b** MCF-7 cells. (Significant differences from control cells are indicated by *p < 0.05, §*p* < 0.01)

The cytotoxic effects of local anesthetics may be due to apoptotic cell death. Significant apoptosis was observed in MDA-MB-231 cells and MCF7 cells treated with six local anesthetics at concentrations higher than 3 mM (Fig. 2). Lidocaine, levobupivacaine, and chloroprocaine at sub-millimolar concentrations (0.3 and 1 mM) also led to significant apoptotic response from MDA-MB-231 cells (Fig. 2a).

### Viability of breast cancer and non-cancer cells treated with local anesthetics at plasma concentrations

**Fig. 3:**
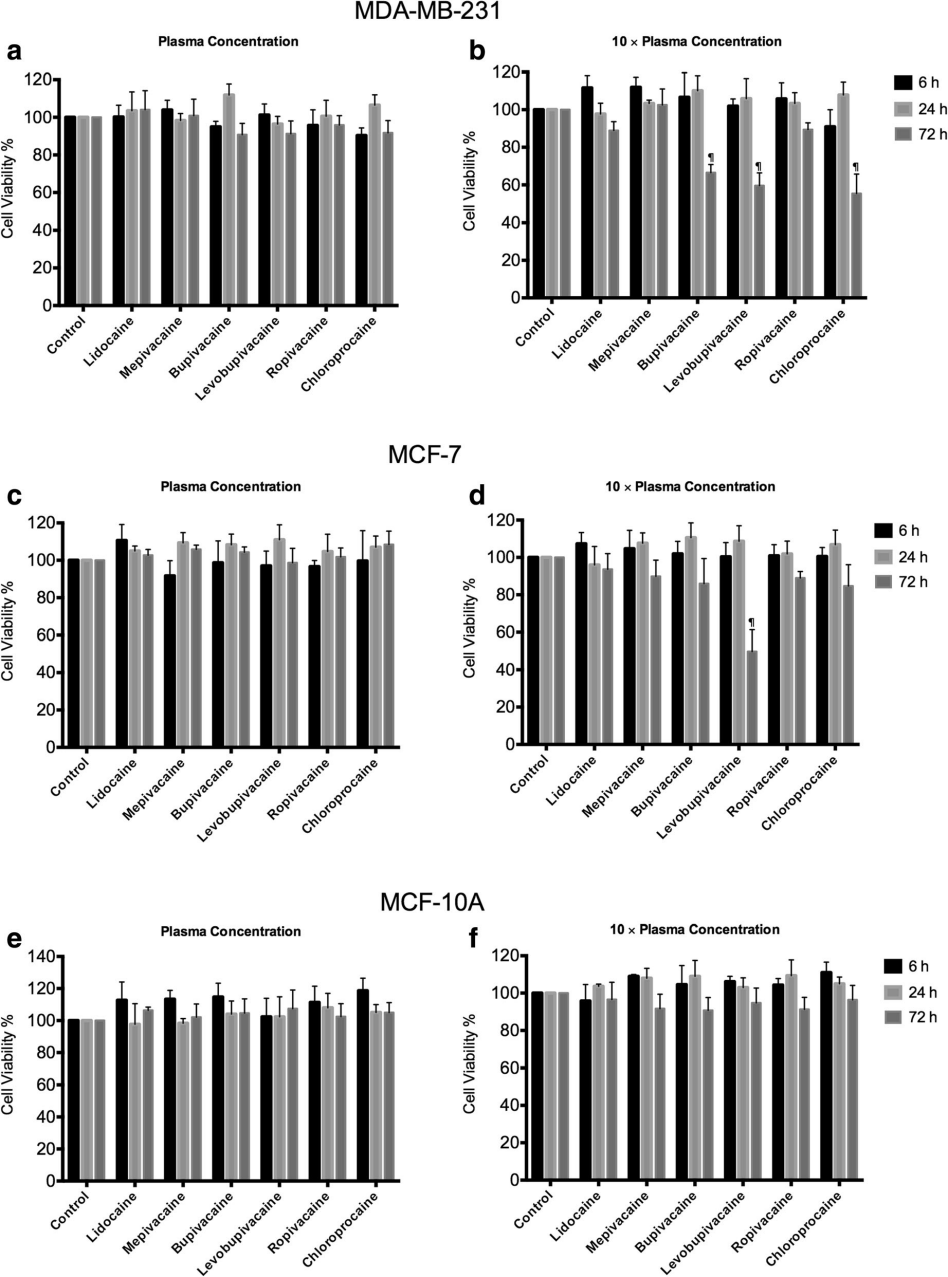
The effect of 1× and 10× plasma concentrations of local anesthetics on the viability of **a**, **b** MDA-MB-231 cells, **c**, **d** MCF-7 cells, and **e**, **f** MCF10A cells. Viability was measured by the MTT assay after 6 h, 1 day or 3 days exposure to the indicated local anesthetic. (Significant differences from control cells are indicated by ¶ *p* < 0.001)

We employed the MTT assay to assess the viability of cells exposed to plasma concentrations local anesthetics. These are concentrations that correspond to plasma levels achieved during anti-arrhythmia treatment with lidocaine or nerve block with the other local anesthetics. Along with MDA-MB-231 and MCF7 breast cancer cells, we included the non-cancerous MCF10A breast cells. These plasma concentrations, applied for up to three days, did not affect the viability of any of the cells (Fig. 3a). For a three-day exposure to 10 × plasma concentrations, however, bupivacaine, levobupivacaine, and chloroprocaine each dramatically inhibited the viability of MDA-MB-231 cells (Fig. 3a). Levobupivacaine, applied at 10 × plasma concentrations for three-days, inhibited the viability of MCF7 cells by more than 50% (Fig. 3b). In contrast to their effects on the two cancer cell lines, local anesthetics at higher concentrations did not affect the viability of MCF10A cells (Fig. 3c). Therefore, local anesthetics selectively inhibit these breast cancer cells over the non-tumorigenic cells.

### Migration of MDA-MB-231 and MCF-7 cells treated with local anesthetics at plasma concentrations

**Fig. 4:**
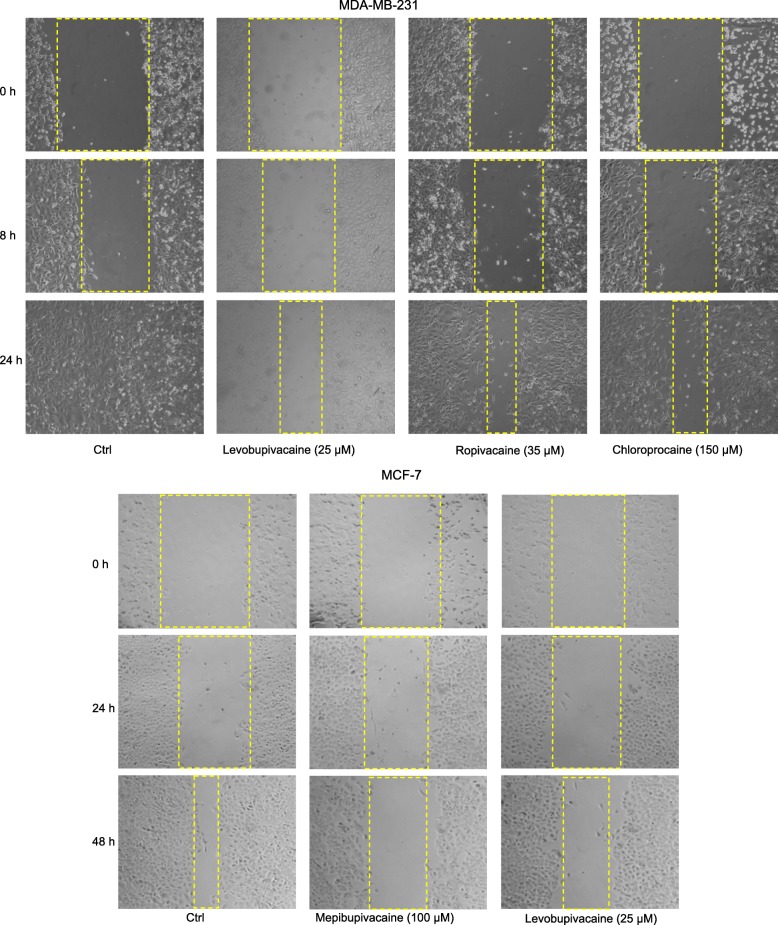
Representative images from the wound-healing assay of MDA-MB-231 and MCF-7 cells treated with local anesthetics. Yellow lines indicate the width of the wound at different times

**Fig. 5:**
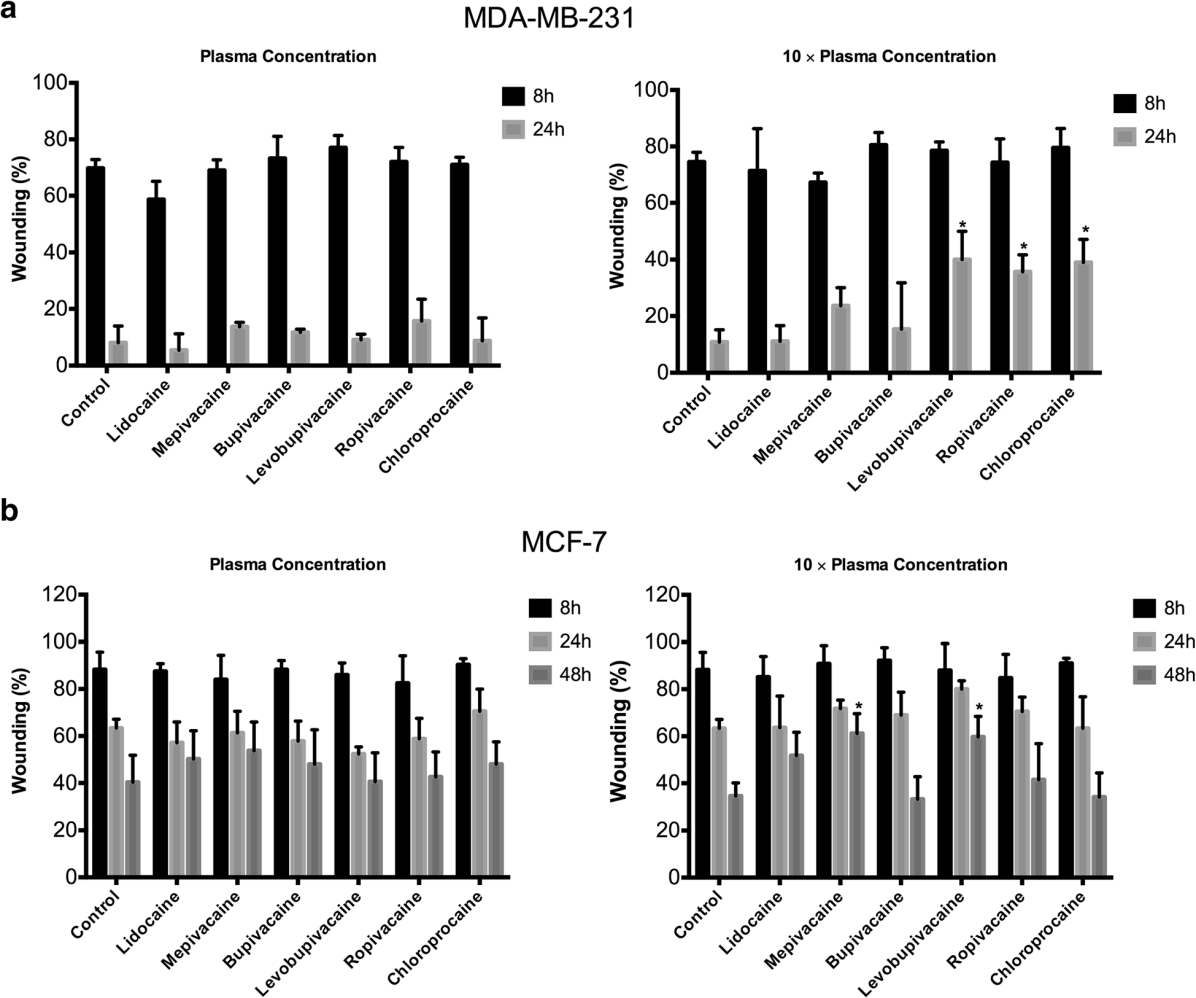
Effect of local anesthetics of plasma and 10x plasma concentrations on migration of breast cancer cells. Wound healing assay showing inhibition of cell migration after 24 h and 48 h of exposure to local anesthetics. **a** In MDA-MB-231 cells, 10x plasma concentrations of ropivacaine, levobupivacaine and chloroprocaine produced significant inhibition. **b** In MCF-7 cells, 10x plasma concentration of mepivacaine, and levobupivacaine produced significant inhibition. (*p < 0.05)

Having established that plasma concentrations of local anesthetics have no effect on cell viability, we used a wound-healing assay to determine whether these concentrations affect cell migration. Representative images are shown in Fig. 4. Because MCF7 cells grow more slowly than MDA-MB-231 cells, we used different measurement time points. At plasma concentrations, none of the six local anesthetics affected the migration of either MDA-MB-231 or MCF7 cells (Fig. 5). At 10 × plasma concentrations, levobupivacaine, ropivacaine, and chloroprocaine significantly inhibited the migration of MDA-MB-231 cells after 24-h exposure (Fig. 5a). Similarly, mepivacaine and levobupivacaine significantly inhibited the migration of MCF7 cell after 48-h exposure (Fig. 5b).

### Cell cycle analysis of MDA-MB-231 cells treated with local anesthetics at plasma concentrations

**Fig. 6:**
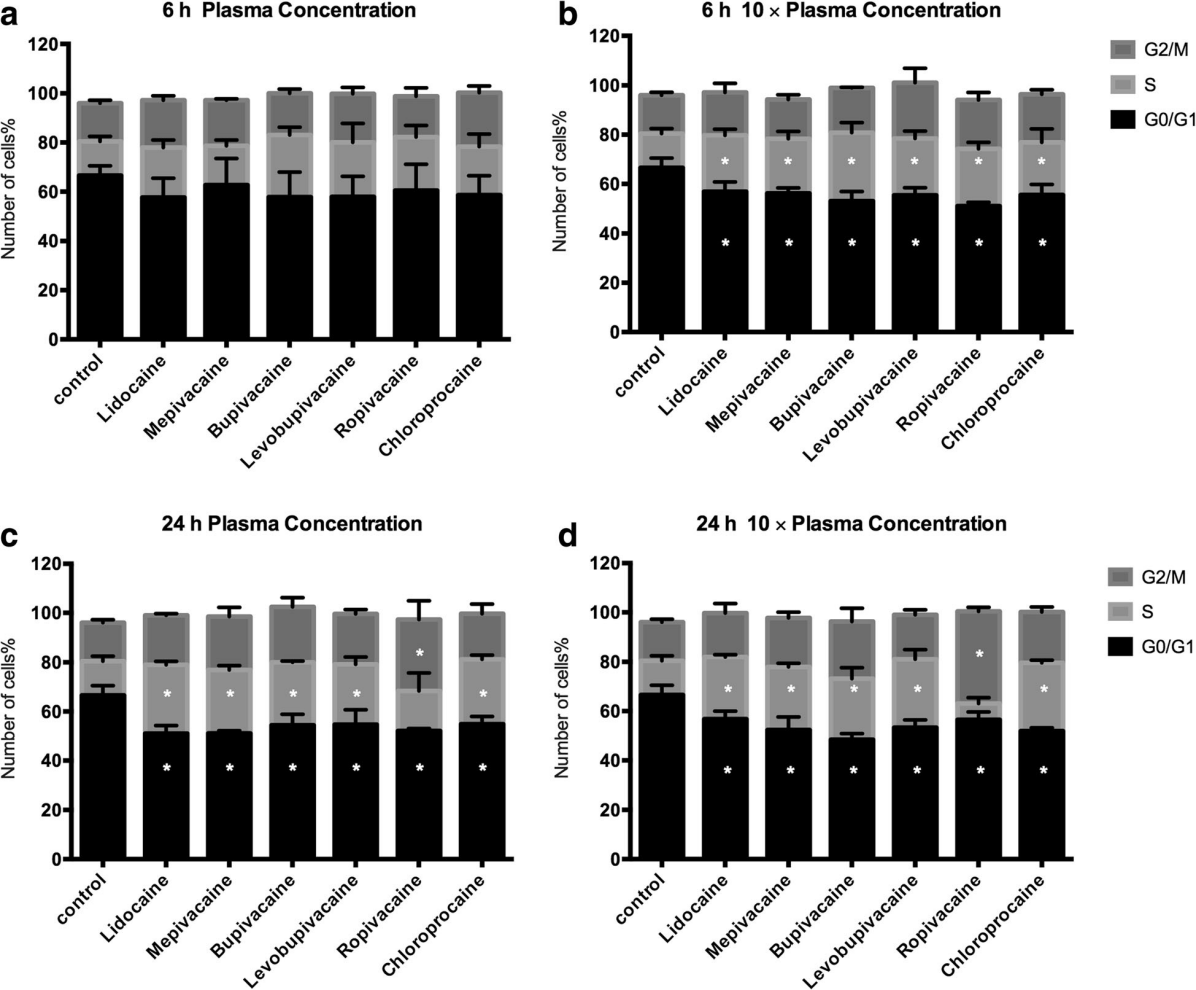
Cell cycle analysis of MDA-MB-231 cells under treatments with different local anesthetics. **a** Six local anesthetics at plasma concentration did not significantly affect cell cycle of MDA-MB-231 cells after 6-h exposure. **c** However, after 24 h, local anesthetics increased cell population of S or G2/M, while decrease cell population of G0/G1. Local anesthetics at 10 × plasma concentration significantly increased S or G2/M phase after 6-h (**b**) and 24-h (**d**) treatments

**Fig. 7:**
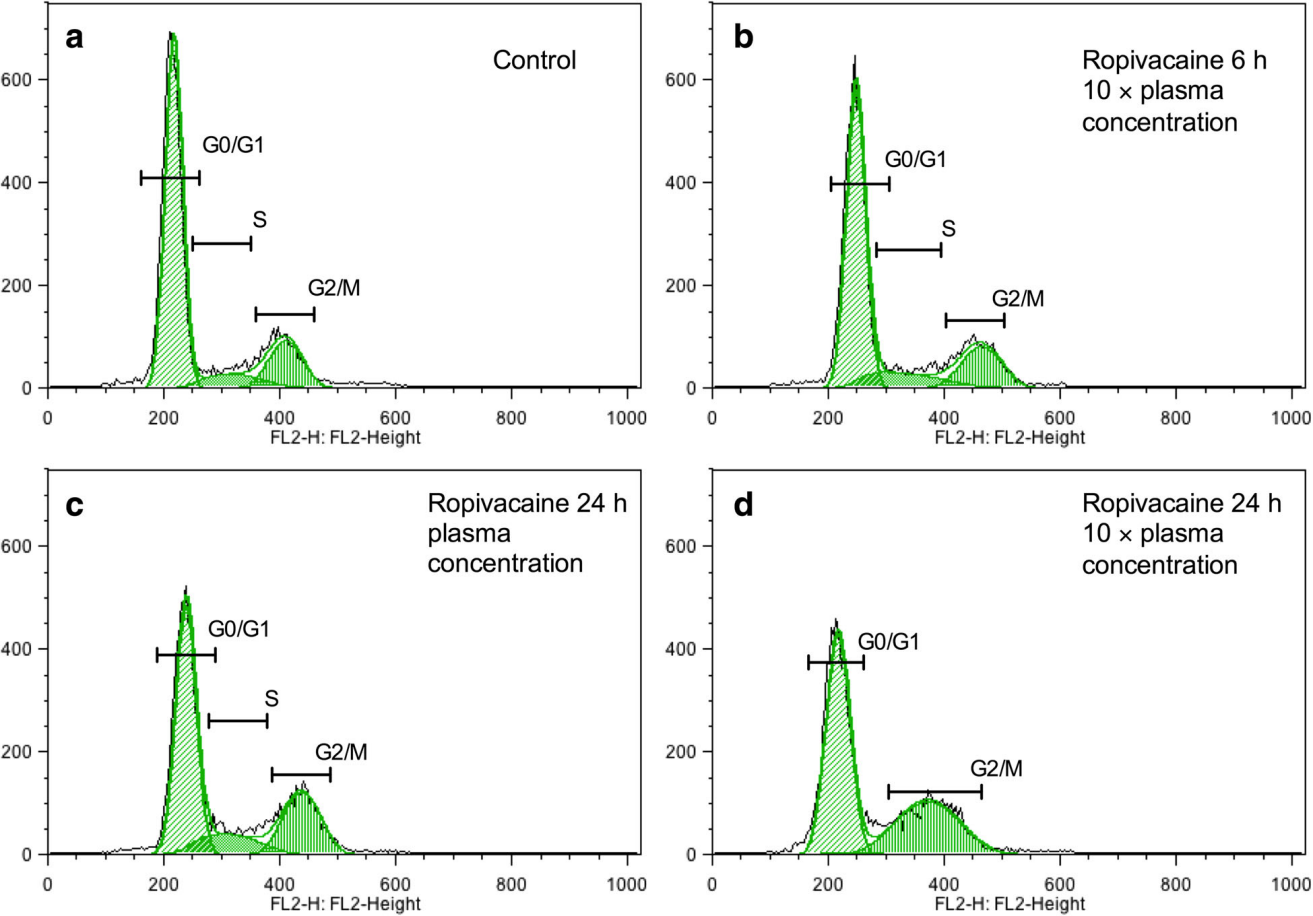
Ropivacaine at 10 × plasma concentration arrested MDA-MB-231 cells at G2 phase after 24-hour incubation. In comparison with control (**a**), ropivacaine at 10 × plasma concentration increased the cell population of S and G2 phase in MDA-MB-231 cells after 6-hour incubation (**b**), and further increased G2 phase after 24-hour incubation (**d**). Ropivacaine at plasma concentration (**c**) also increased cell population of S and G2 phase after 24-hour incubation

According to our cell viability and migration data, MDA-MB-231 cells are more sensitive than MCF7 cells to local anesthetics. Thus, our next step was to investigate which stages of the cell cycle are affected by local anesthetics in MDA-MB-231 cells (Fig. 6). After a 6-h exposure to plasma concentrations of local anesthetics, there was no change in the distribution of cells in each phase (Fig. 6a). After 24 h, however, there was an increase in the percentage of cells in the S phase and a corresponding decrease in the G0/1 phase (Fig. 6b). For local anesthetics at 10 × plasma concentrations, the shift from G0/1 to S phase was already seen after 6 h (Fig. 6c) and this persisted after 24 h (Fig. 6d). Interestingly, the 24-h treatment of cells with ropivacaine at 10 × plasma concentration resulted in a drastic enrichment of cells in the G2 phase, suggesting blockade of the cell cycle before mitosis (Fig. 7).

## Discussion

In this study we compared six commonly used local anesthetics at plasma concentrations and above, on breast cancer cell viability, migration, and cell division. This information on the potency and efficacy of local anesthetics may be used as a basis for selecting local anesthetics for study in animal models of cancer and in clinical trials comparing the effects of different types of anesthesia on cancer proliferation.

Previous studies have been limited mostly to lidocaine and bupivacaine, at millimolar concentrations. Here we screened five amide local anesthetics (lidocaine, mepivacaine, levobupivacaine, and ropivacaine) and one ester local anesthetic chloroprocaine. In one study, 4.5 mM lidocaine and 1.3 mM bupivacaine were found to inhibit the viability of MCF-7 cells by inducing apoptosis [12]. A second study found that lidocaine at concentrations higher than 1 mM significantly impaired cell viability of MDA-MB-231 cells, prostatic cancer PC-3 cells, and ovarian cancer ES-2 cells [13]. Another more recent study showed that 5 mM lidocaine or ropivacaine significantly inhibited the growth of human hepatocellular carcinoma through modulation of cell cycle-related genes [14]. Here, we confirmed the direct toxic effects of all tested local anesthetics at millimolar concentrations (1 ~ 10 mM) on breast cancer cells as determined by MTT and LDH assays (Figs. 1 and 2). The clinical preparation of lidocaine for local injection ranges from 0.5% (18.5 mM) to 2% (74 mM). However, the tissue concentrations following local infiltration is difficult to measure. It depends on the speed of injection, the concentration and volume, time of measurement, and the tissue composition and blood supplies. Only a few studies analyzed the tissue concentration of lidocaine. In a recent study using rabbit, the concentration of lidocaine reached peak value in jaw bone (114 μg/g) and oral mucosa tissue (156 μg/g) after 0.5 mL of 2% lidocaine injection for 10 min, which is estimated to be 0.42 mM and 0.58 mM assuming a tissue density of 1 g/ml [15]. This is probably an underestimate for molar concentration since tissues are composed of both “solid” and “soluble” compositions, or cellular and extracellular compartments. It is quite likely, the breast tissue concentrations after local infiltration of 0.5% (18.5 mM) lidocaine range from mini-molar, sub-millimolar and micromolar depending on the time and proximity of injection. Thus, the effects of both 10 × plasma and mini-molar concentrations are clinically relevant and might potentially be beneficial against postoperative metastasis. Currently, there is one ongoing clinical trial with an expected 1600 patient enrollment and an estimated completion date of 2021, testing the effects of local peritumor infiltration with 60 ml of 0.5% (18.5 mM) lidocaine in breast cancer patients (NCT01916317) [2, 16]. It will be interesting to compare the results of this trial with a trial evaluating the effect of intravenous lidocaine on postoperative outcome of patients with breast cancers (NCT01204242) [17].

Although local anesthetics may reach sub-millimolar concentrations at the site of injection, plasma concentrations following regional anesthesia are considerably lower. Among the local anesthetics used clinically, lidocaine is the only local anesthetic that can be administered intravenously at an anti-arrhythmic dose, that is, a plasma concentration of 5–20 μM [7, 8]. The plasma concentration after regional anesthesia with lidocaine is in a similar range [18]. Lidocaine at this dose has been used in several “innovative” ways. For example, it has been used for neuroprotection in cardiac surgery patients [19, 20], for reduction of opiate usage in ambulatory surgery patients [21], and for reduction of postoperative ileus and pain following colon resection [22]. It would be very attractive if this intravenous level of lidocaine could suppress the viability and motility of circulating cancer cells. However, we did not detect any significant effects of lidocaine (or any other local anesthetic) in this dose range. The plasma concentration of lidocaine effectively blocks neuronal voltage gated sodium channels [23], but this does not apply to cancer cells. However, with 3-day treatments at 10 times of plasma concentration, we found that some local anesthetics, particularly levobupivacaine and chloroprocaine, directly inhibited viability of both breast cancer cell lines MDA-MB-231 and MCF-7 (Fig. 3), but not the non-cancerous breast epithelial cell line MCF-10A. Although lidocaine is more widely studied among other local anesthetics, our results suggest that levobupivacaine induced a more potent reduction of cell viability than other local anesthetics on breast cancer cells. Similarly, Jose et al. has demonstrated a strong cytotoxic effect of levobupivacaine on cancer cell viability through inhibiting mitochondrial energy production [24]. Moreover, we observed that triple negative breast cancer cells (MDA-MB-231) are more sensitive than estrogen receptor-positive breast cancer cells (MCF-7) in response to local anesthetics, which indicate a cell-type specific effect.

Inhibition of cell migration is another way in which local anesthetics might affect cancer cells. It has been reported that 1 mM lidocaine inhibited the invasion and migration of MDA-MB-231 cells, prostatic cancer PC-3 cells, and ovarian cancer ES-2 cells [13]. We did not find any significant direct effects of lidocaine on breast cancer migration at the plasma concentration (10 μM). However, mepivacaine, levobupivacaine, ropivacaine, and chloroprocaine significantly inhibited the migration of MDA-MB-231 and/or MCF-7 at 10 times of plasma concentration (Fig. 4).

To further explore the effects of local anesthetics at plasma concentrations on breast cancer cell function, we looked for changes in the cell cycle in MDA-MB-231 cells. The cell cycle and cell growth are tightly regulated in normal cell, but genomic and epigenetic dysregulation lead to the uncontrolled proliferation of cancer cells. Few studies have investigated the effect of local anesthetics on the cell cycle. Le Gac and colleagues analyzed lidocaine and ropivacaine on human hepatocellular carcinoma cells. They found that 100 μM ropivacaine arrested the cell cycle at the G2 phase, whereas 100 μM lidocaine had little effect. They also observed that ropivacaine selectively modulated the expression of key cell cycle-related genes [14]. In our study, 24-h treatment with any of the six local anesthetics at plasma concentration or 10 times of plasma concentration led to an increase in cells in S phase and a decrease in G0/G1 (Fig. 7a). This indicates an arrest in the cell cycle process from S (DNA replication) phase to G2/M phase, and may result in arresting mitosis and cellular apoptosis. Consistent with the above study of human hepatocellular carcinoma, ropivacaine at 35 μM increased the percentage of cells in the G2 phase, which may attribute to the blockage of cell cycle from G2 (preparation for cell division) to M (cell division). Further research is needed to examine the detail mechanism of cell cycle arrest in local anesthetic-treated breast cancer cells.

The potential beneficial effects of using local anesthesia during cancer surgery include attenuating surgical stress from neuroendocrine disturbance that promote the development of metastasis [25], reducing usage of systemic anesthesia and opiates [26], which inhibit cell-mediated immunity, and a direct effect on the cancer cells. Our results show that it is difficult to delineate a common mechanism to account for the direct inhibition of cancer cell growth by all the tested local anesthetics. We have shown that different local anesthetics may exert differential effects by various mechanisms in cancer cells. For example, levobupivacaine and chloroprocaine clearly exhibited anti-proliferation and anti-migration effect on breast cancer cells, while ropivacaine affects the cell cycle of breast cancer cells. Moreover, the two breast cancer cell lines we employed in this study displayed differential responses to local anesthetics. This indicates that heterogeneity of breast cancer may play an important role in determining the usefulness of local anesthetics on decreasing cancer recurrence. Therefore, future mechanistic studies must focus on specific breast cancer subtypes. Although, clinical trials point towards beneficial effects of local anesthetics on cancer metastasis; we found that plasma concentrations of a variety of local anesthetics had no significant effects on the viability and migration of two subtypes of breast cancer cells. It may be that local anesthetics affect cancer metastasis through modulation of the tumor microenvironment. This will be the direction of our future studies.

## Conclusion

The effects of local anesthetics on viability and migration of breast cancer cells depends on the concentration of individual anesthetic, the duration of exposure and the cell line. Levobupivacaine at 10 times of plasma concentration have a higher potency over other local anesthetics reducing the viability and migration of breast cancer cells. Ropivacaine at 10 times of plasma concentration arrests breast cancer cell cycle in G2 phase. Overall, we demonstrated that local anesthetics at high concentrations significantly affect breast cancer cell function.
